# Integrating graph neural networks and LSTM for path optimization in smart port multi-modal systems

**DOI:** 10.1371/journal.pone.0336629

**Published:** 2025-12-02

**Authors:** Jiangjiang He, Weixun Chen, Jiaren Sun, Lin Zhu

**Affiliations:** 1 School of Transportation and Logistics, Guangzhou Railway Polytechnic, Guangzhou, China; 2 School of Information Engineering, Guangzhou Railway Polytechnic, Guangzhou, China; King Khalid University, SAUDI ARABIA

## Abstract

This paper addresses the challenges of dynamic environments and multimodal data fusion in multimodal transport path optimization for smart ports by proposing a GL-SSL Model that integrates Graph Neural Networks (GCN), Long Short-Term Memory (LSTM), and Self-Supervised Learning (SSL). The model fully exploits the graph-structured information of port transport networks and their temporal variations, while SSL enhances feature representation, enabling efficient optimization of path planning. Experiments were conducted on multiple public datasets, including AIS data from the Port of Rotterdam, global shipping data, and port net revenue data. Results show that the GL-SSL Model achieved significant improvements in key performance metrics. Specifically, the optimized path length reached **80 km**, the transport cost was reduced to **200 cost-units** (a composite metric reflecting fuel consumption, equipment wear, and labor cost), and the delay rate was maintained at **0.05 (5%)**, all of which are substantially better than traditional algorithms and other deep learning models. Furthermore, the model demonstrated stable performance under complex scenarios such as peak traffic, adverse weather, and equipment failures, with rapid convergence of training loss and strong robustness. These findings highlight the model’s adaptability and practical application potential. Overall, this work provides effective technical support for multimodal transport path optimization in smart ports and carries important theoretical significance and broad application prospects.

## Introduction

In the context of the continuous growth in global trade volumes and the intelligent transformation of logistics, smart ports have become a central hub in the modern logistics system, handling over 90% of global cargo traffic. The operational efficiency of ports directly influences the speed and cost of international trade circulation [1]. Port multimodal transportation, through the integration of maritime, railway, and road transport modes, has significantly improved cargo turnover efficiency but also faces unprecedented challenges in path optimization [2,3]. The port transportation system involves complex topological relationships between spatial entities such as ports, docks, and waterways, and must also respond in real-time to uncertainties in tidal changes, fluctuations in ship arrival times, and traffic congestion, while also accounting for seasonal and unexpected changes in transportation demand [4]. Traditional static path planning methods are inadequate for addressing the dynamic decision-making needs of these systems.

Existing path optimization methods have significant limitations. Traditional heuristic algorithms, such as Dijkstra’s and genetic algorithms, rely on fixed cost functions and static network parameters, making it difficult to capture the dynamic spatiotemporal characteristics of port transportation [5,6]. Deep learning-based methods, while having made breakthroughs in time-series forecasting, still exhibit notable shortcomings. For instance, convolutional neural networks (CNNs) struggle to effectively capture the complex topological relationships between nodes in non-Euclidean structured data, such as port networks [7,8]. Recurrent neural networks (RNNs) and their variants can handle sequential data, but when capturing the long-range dependencies of port transportation demands, they are prone to gradient disappearance or explosion, leading to reduced prediction accuracy [9–11]. Graph neural networks (GNNs) can effectively aggregate node and neighbor information through graph convolution operations, demonstrating unique advantages in processing the spatial topological structure of port networks, but single GNN models struggle to address dynamic changes in temporal dimensions such as transportation demands and traffic flows [12,13]. Long short-term memory networks (LSTMs) solve the long-range dependency problem of traditional RNNs through gating mechanisms and perform excellently in time-series prediction, yet they cannot fully utilize the spatial structure information of port networks [14].Self-Supervised Learning (SSL) addresses the shortage of labeled data in port transportation by extracting features from large unlabeled datasets through pretraining tasks like node and edge reconstruction, and contrastive learning, which, when integrated with GCN and LSTM, enhances learning efficiency and spatiotemporal optimization [15,16].

Building on these insights, this study introduces the GL-SSL Model—an innovative framework for multimodal path optimization in smart ports that integrates GCN, LSTM, and SSL. The model’s key innovation lies in combining GCN for capturing spatial topologies of the port network with LSTM for modeling long-term temporal dynamics in transportation demand and traffic flow. Additionally, SSL leverages large-scale unlabeled data to uncover latent features, significantly improving the model’s generalization capabilities.

The main contributions of this research are as follows:

- A multimodal path optimization method combining graph-based modeling and time-series forecasting to accurately characterize the spatiotemporal dynamics of port operations.
- The use of a self-supervised learning mechanism to reduce reliance on labeled data and improve the model’s adaptability in complex environments.
- Through validation on real-world port datasets, the model significantly outperforms traditional methods in path optimization efficiency, resource utilization, and dynamic responsiveness, providing theoretical and technical support for the intelligent upgrading of smart ports.

The subsequent sections of this paper will systematically discuss the GL-SSL Model: first, a review of the related research in the field of smart port path optimization will be presented, analyzing the progress and limitations of existing technologies; second, the model’s architecture design, algorithmic process, and parameter optimization strategies will be detailed, highlighting the technical intricacies of integrating graph structure and time-series modeling; then, based on multiple real-world port datasets, comprehensive experiments and comparative analyses will be conducted to evaluate the model’s performance in terms of path optimization accuracy and dynamic responsiveness; finally, the research outcomes will be summarized, and the application value and future directions for improvement will be explored.

## Related works

### Port path optimization methods

**Fig 1:**
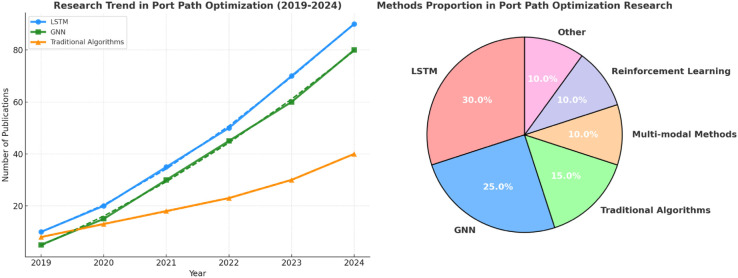
Port Path Optimization Research Trends and Method Proportions from 2019 to 2024: Literature Growth and Distribution of Different Optimization Methods.

In recent years, research in the field of port path optimization has experienced rapid development, with the focus gradually shifting from traditional algorithms to deep learning and multimodal fusion technologies. With the growth of global trade and the advancement of smart port construction, traditional static optimization methods have become increasingly inadequate to meet the complex and changing demands of port transportation, prompting researchers to explore more efficient and intelligent path optimization strategies. Based on bibliometric visual analysis (Fig 1), the research interest in deep learning methods has been steadily rising, and multimodal fusion technologies have become a major research trend in recent years, reflecting the development trend towards dynamic and intelligent solutions in this field. Especially in recent years, with the combination of graph neural networks (GNN) and deep learning, the research on port path optimization has gradually shown a new trend. Graph neural networks have been applied to a variety of dynamic network modeling, showing their strong potential in complex transportation systems [17,18].

As shown in Table 1, all current methods have significant limitations. Traditional algorithms such as Dijkstra and A* algorithms, although mature in theory and simple in computational logic, have low computational efficiency and lack adaptability when faced with large-scale port networks and dynamic traffic changes; among deep learning methods, LSTM and GRU are good at processing time series, but the computational cost is high when dealing with large-scale data, while graph processing models such as GNN and GCN are difficult to take into account dynamically changing time dimension information [19–21]. For example, in recent years, graph convolutional networks (GCN) and graph attention networks (GAT) have been widely used in the modeling and path optimization of port transportation networks, but they still have large computational and storage overheads when faced with large-scale graph structures [22,23]. Multimodal fusion methods such as CNN+LSTM and CNN-GNN can integrate different types of data, but the model complexity is high, the training time is long, and the actual deployment is difficult [24]. In addition, reinforcement learning related methods (such as RL and Q-learning) have the problems of slow convergence speed and large data requirements, and the complexity of task design of self-supervised learning (SSL) also restricts its practical application effect. Self-supervised learning (SSL) provides a new approach for learning unlabeled data. In recent years, path optimization methods based on SSL have gradually become a research hotspot [25].

**Table 1:** Comparison of Different Port Path Optimization Methods: Traditional Methods, Deep Learning Methods, and Multimodal Fusion Methods, Highlighting Their Advantages, Disadvantages, and Application Scenarios.

Method	Advantages	Disadvantages	Application Scenarios
Dijkstra Algorithm [26,27]	Simple to compute, well-established theory	High time complexity, unable to handle dynamic changes	Small-scale ports, static path optimization
A* Algorithm [28,29]	Efficient in finding the shortest path, suitable for larger networks	High computational cost for large-scale networks	General path optimization, static traffic flow
LSTM [30]	Captures long-term dependencies in time-series, suitable for dynamic data	Low efficiency for large-scale data, high computational cost	Dynamic transportation demand prediction, vessel scheduling
GRU [31,32]	More efficient than LSTM, suitable for time-series data	May not perform as well as LSTM in long-sequence prediction	Time-series modeling, traffic flow prediction
GNN [33–35]	Suitable for graph-structured data, can capture spatiotemporal relationships between nodes	High computational and storage costs for large-scale graphs	Port transportation network modeling, path optimization
GCN [36,37]	Efficient for processing graph data, can handle complex dependencies between nodes	High computational cost for large-scale graphs, difficult to handle dynamic changes	Port network modeling, path optimization
GAT [38,39]	Enhances performance by assigning different weights to neighboring nodes via self-attention	High computational and memory costs, difficult to scale to very large graphs	Graph data optimization, port transportation network optimization
CNN + LSTM [40,41]	Combines image data analysis and time-series data modeling to optimize path planning	High complexity in processing large-scale data, long training time	Image data analysis, time-series analysis, port transportation management
RL [42]	Adaptive learning, dynamically adjusts path planning strategies	Low efficiency for large-scale environments, slow convergence	Dynamic path optimization, resource scheduling
Q-learning [43–45]	Finds the optimal path through value iteration	High computational and memory costs for large-scale problems, poor offline learning performance	Small-scale path optimization, static network models
SSL [46]	No need for labeled data, learns data features through pre-tasks	Complex task design, difficult to ensure model effectiveness	Path optimization with unlabeled data, graph structure learning
Transformer-LSTM [47,48]	Can process both graph-structured and time-series data, suitable for multimodal tasks	Long training time, high computational cost	Multimodal path optimization, demand prediction, traffic flow management
CNN-GNN [49]	Combines image features with graph-structured data, improving path planning and optimization performance	Complex image data preprocessing, high computational cost	Port image analysis, transportation route optimization
RL-LSTM [50,51]	Combines the adaptive learning ability of RL with time-series modeling of LSTM to adapt to dynamic changes	Slow convergence, high training data requirements	Dynamic path optimization, resource scheduling, time-series prediction

Current research has yet to establish an effective method that can comprehensively integrate the spatial topological features of port transportation networks, dynamic temporal changes, and overcome the issue of insufficient labeled data. To address this gap, this study proposes the GL-SSL Model, which innovatively integrates Graph Convolutional Networks (GCN), Long Short-Term Memory (LSTM) networks, and Self-Supervised Learning (SSL). The model aims to overcome the limitations of traditional path optimization methods and achieve efficient dynamic optimization of multimodal port transport paths for smart ports.

Existing research has not yet developed an effective method that can fully integrate the spatial topological characteristics and time series dynamic changes of the port transportation network while overcoming the problem of insufficient labeled data [52]. To this end, this study proposed the GL-SSL Model, which innovatively integrates graph convolutional networks (GCNs), long short-term memory networks (LSTMs) and self-supervised learning (SSL) to achieve efficient dynamic optimization of port transportation network paths, overcoming the bottlenecks of traditional path optimization methods. It is particularly innovative in multi-source heterogeneous data fusion and large-scale dynamic network modeling.

## Methodology

### Overall of GL-SSL model

**Fig 2:**
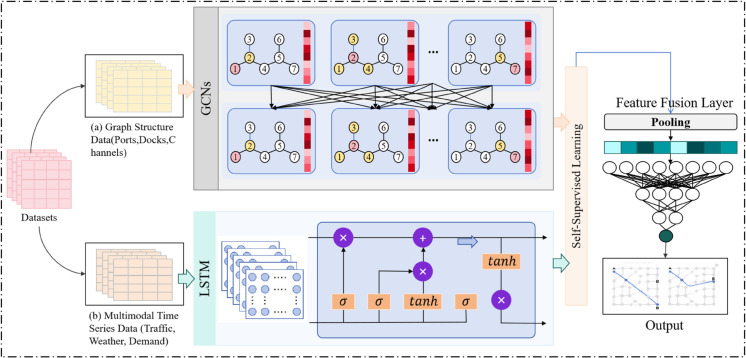
Overall Architecture of the GL-SSL Model for Smart Port Path Optimization, Integrating GCN, LSTM, and SSL for Efficient Multimodal Path Planning and Dynamic Decision-Making in Port Transportation Networks.

The GL-SSL Model (Graph-LSTM with Self-Supervised Learning for Smart Port Path Optimization) proposed in this study aims to achieve efficient optimization of multimodal port intermodal paths by integrating Graph Convolutional Networks (GCN), Long Short-Term Memory Networks (LSTM), and Self-Supervised Learning (SSL). The overall model architecture is shown in Fig 2. The core design concept revolves around the spatiotemporal characteristics of the port transportation system. Through the collaborative operation of multiple modules, the model deeply integrates the spatial topology of the port network with the dynamic temporal changes in the transportation process, while utilizing SSL to enhance the model’s ability to extract features from unlabeled data.

At the input layer, the model receives two types of core data: graph-structured data and multimodal time-series data. The former abstracts entities such as ports, docks, and waterways as nodes, and relationships such as transport routes, distances, and throughput as edges, forming a graph structure of the port transportation network. The latter includes time-varying data such as transportation demand predictions, vessel and vehicle dynamics, and environmental factors (e.g., weather, traffic flow). These data types serve as the input basis for GCN and LSTM, providing rich information sources for subsequent modeling.

In the core processing modules of the model, GCN and LSTM perform distinct yet collaborative functions. The GCN module (left side of Fig 2) deeply models the graph structure of the port transportation network through propagation operations on the adjacency matrix and node feature matrix. Specifically, through multiple layers of graph convolution operations, GCN aggregates the features of each node with information from its neighboring nodes, progressively learning spatial dependencies from local to global, and outputting node representations that contain spatial topological features. Meanwhile, the LSTM module (right side of Fig 2) handles multimodal time-series data, capturing long-term dependencies in dynamic changes such as transportation demand and traffic flow through gating mechanisms. It predicts future transportation trends and environmental changes. The spatial features output by GCN and the temporal features extracted by LSTM are then fused, forming a comprehensive feature representation with both spatial and temporal dimensions, providing a more holistic basis for path optimization decisions.

The Self-Supervised Learning (SSL) module acts as the “enhancement engine” of the model, embedded in the training process of both GCN and LSTM through pre-task mechanisms. For example, reconstruction tasks for nodes and edges, as well as neighbor node prediction tasks based on graph data, are designed. A contrastive learning strategy is employed to explore the feature differences of port graphs at different time points, helping the model learn underlying patterns from large amounts of unlabeled data (shown in the middle of Fig 2). This self-supervised learning approach not only improves the model’s efficiency in learning spatiotemporal features of the port but also significantly enhances its generalization ability in complex and dynamic scenarios.

At the output layer, the model uses the fused spatiotemporal features to perform path planning and optimization decisions. By comprehensively considering multiple objective constraints such as transportation time, cost, and resource utilization, the model outputs the optimal path solution for smart port multimodal transport. Additionally, relying on LSTM’s ability to predict future dynamic changes, the model can monitor environmental changes in real time, dynamically adjust path selection, and coordinate the scheduling of port transportation resources (e.g., vessels, vehicles, storage), ensuring the efficient operation of the transportation system. The entire model architecture, through the organic collaboration of multiple modules, provides a systematic modeling and dynamic solution for the port multimodal path optimization problem, offering an innovative solution for the intelligent operation of smart ports.

### GCN module

**Fig 3:**
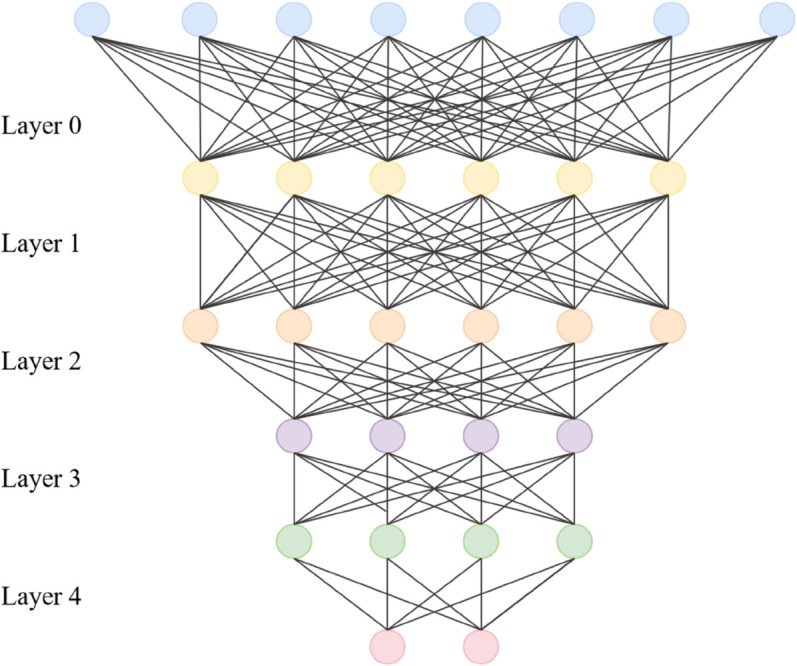
Architecture of the GCN Module in the GL-SSL Model for Smart Port Path Optimization, Illustrating the Aggregation of Spatial Topological Features from Port Transportation Networks Using Node and Edge Feature Matrices, Node Type Embedding, and Edge Feature Enhancement.

In the GL-SSL Model, the Graph Convolutional Network (GCN) [36] module serves as the core component for processing the spatial structure of the port transportation network. Its design is closely aligned with the complex topological relationships between nodes and edges in the port multimodal transportation scenario (Fig 3). As mentioned earlier, the port transportation network includes various entities such as ports, docks, waterways, and warehouses, as well as relational attributes like transport routes, throughput capacity, and distance costs. These factors form a non-Euclidean structure, which is the specialty of GCN.

The GCN module takes as input the adjacency matrix $𝐀\in {\mathbb{R}}^{N\times N}$ and the node feature matrix $𝐗\in {\mathbb{R}}^{N\times F}$, where *N* is the number of nodes in the network and *F* is the initial feature dimension of each node. To effectively aggregate spatial information between nodes, GCN uses a normalized graph convolution operation. The feature update equation for a single layer of GCN is as follows:

$${𝐇}^{\left(l+1\right)}=\sigma \left({\tilde{𝐃}}^{-\frac{1}{2}}\tilde{𝐀}{\tilde{𝐃}}^{-\frac{1}{2}}{𝐇}^{\left(l\right)}{𝐖}^{\left(l\right)}\right)$$ (1)

where ${𝐇}^{\left(l\right)}$ represents the node feature matrix at the *l*-th layer, and initially, ${𝐇}^{\left(0\right)}=𝐗$. ${𝐖}^{\left(l\right)}$ is the learnable weight matrix at the *l*-th layer, used to extract key information from the node features. $\tilde{𝐀}=𝐀\;+\;𝐈$ is the adjacency matrix with self-loops added, ensuring that each node retains its own feature information. $\tilde{𝐃}$ is the diagonal node degree matrix of $\tilde{𝐀}$, with elements ${\tilde{𝐃}}_{ii}=\sum_{j}{\tilde{𝐀}}_{ij}$, which is used to normalize the adjacency matrix, preventing numerical instability during training. *σ* is the activation function, and in this study, we use the ReLU (Rectified Linear Unit) function, i.e., σ(x)=max(0,x), to enhance the model’s non-linear expressive power.

In the smart port scenario, different types of nodes (e.g., docks, warehouses) have varying weights in their influence on path planning. To capture this heterogeneity, this study introduces a node type embedding mechanism into the basic GCN structure. Let the node type matrix $𝐓\in {\mathbb{R}}^{N\times T}$, where *T* is the node type dimension, and by merging the node type embedding with the feature matrix, Equation (1) can be extended as:

$${𝐇}^{\left(l+1\right)}=\sigma \left({\tilde{𝐃}}^{-\frac{1}{2}}\tilde{𝐀}{\tilde{𝐃}}^{-\frac{1}{2}}({𝐇}^{\left(l\right)}{𝐖}^{\left(l\right)}+𝐓{𝐄}^{\left(l\right)})\right)$$ (2)

where ${𝐄}^{\left(l\right)}$ is the type embedding weight matrix at the *l*-th layer. Through this mechanism, the GCN can better learn the spatial dependencies of different types of nodes in path optimization.

Furthermore, considering that edge attributes in the port transportation network (e.g., transportation cost, travel time) are crucial for path decision-making, this study introduces an edge feature enhancement mechanism. Let the edge feature matrix be ${𝐄}_{f}\in {\mathbb{R}}^{N\times N\times F_{e}}$, where *F*_*e*_ is the edge feature dimension. The edge features are incorporated into the node feature update process through weighted summation:

$${𝐇}^{\left(l+1\right)}=\sigma \left({\tilde{𝐃}}^{-\frac{1}{2}}(\tilde{𝐀}⊙{𝐄}_{f}){\tilde{𝐃}}^{-\frac{1}{2}}{𝐇}^{\left(l\right)}{𝐖}^{\left(l\right)}\right)$$ (3)

where ⊙ represents element-wise multiplication. With the improvements in Equations (2) and (3), the GCN module can capture the spatial topological features of the port transportation network more comprehensively, providing a more accurate spatial representation for subsequent fusion with LSTM’s spatiotemporal features.

By stacking *L* layers of GCN, the model can progressively aggregate node information from local to global. This process is clearly depicted in Fig 3. Shallow GCN layers focus on capturing first-order neighborhood relationships (such as the transport capacity between adjacent docks), while deeper layers integrate higher-order neighbor information (such as the global connectivity of port networks across regions). The final output, ${𝐇}^{\left(L\right)}$, contains rich spatial semantics and serves as the critical spatial feature foundation for multimodal path optimization.

### LSTM module

**Fig 4:**
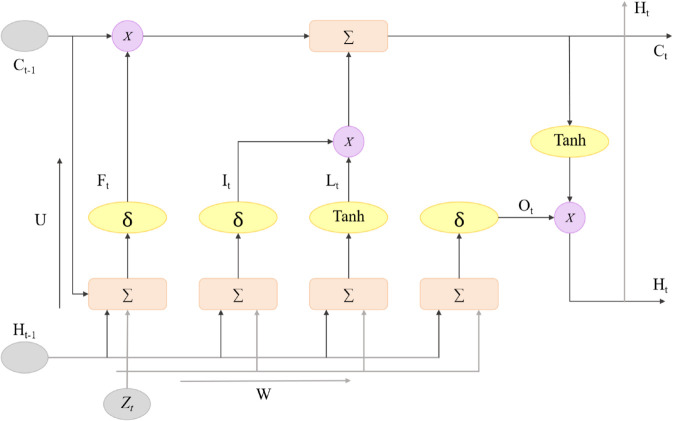
Architecture of the LSTM Module in the GL-SSL Model for Smart Port Path Optimization, Capturing Temporal Dependencies and Integrating Attention Mechanism for Dynamic Path Planning and Decision-Making in Port Transportation Systems.

In the GL-SSL Model, the Long Short-Term Memory (LSTM) [30] module is the core unit for capturing the dynamic temporal changes in the port transportation system. Its architecture is designed to address the complexity of multimodal time-series data (as shown in Fig 4). The port transportation process involves multi-dimensional time-series information, such as fluctuations in transportation demand, variations in vessel arrival times, and traffic flow changes. These data exhibit significant long-term dependencies and nonlinear patterns that traditional Recurrent Neural Networks (RNNs) struggle to handle effectively.

The LSTM module takes as input multimodal time-series data $𝐗=[{𝐱}_{1},{𝐱}_{2},\ldots ,{𝐱}_{T}]$, where ${𝐱}_{t}\in {\mathbb{R}}^{M}$ represents the input vector at time step *t*, and *M* is the feature dimension, encompassing multimodal information such as cargo transportation volume, vessel real-time locations, weather conditions, and other relevant data. The core operations of the LSTM unit are controlled by the forget gate *f*_*t*_, input gate *i*_*t*_, cell state ${𝐜}_{t}$, and output gate *o*_*t*_, with the update process described in the following equations:

$$f_{t}=\sigma ({𝐖}_{f}\cdot [{𝐡}_{t-1},{𝐱}_{t}]+{𝐛}_{f})$$ (4)

$$i_{t}=\sigma ({𝐖}_{i}\cdot [{𝐡}_{t-1},{𝐱}_{t}]+{𝐛}_{i})$$ (5)

$${\tilde{𝐜}}_{t}=\tanh({𝐖}_{c}\cdot [{𝐡}_{t-1},{𝐱}_{t}]+{𝐛}_{c})$$ (6)

$${𝐜}_{t}=f_{t}⊙{𝐜}_{t-1}+i_{t}⊙{\tilde{𝐜}}_{t}$$ (7)

$$o_{t}=\sigma ({𝐖}_{o}\cdot [{𝐡}_{t-1},{𝐱}_{t}]+{𝐛}_{o})$$ (8)

$${𝐡}_{t}=o_{t}⊙\tanh({𝐜}_{t})$$ (9)

where ${𝐡}_{t-1}$ and ${𝐜}_{t-1}$ represent the hidden state and cell state at time step *t*–1, respectively. ${𝐖}_{f},{𝐖}_{i},{𝐖}_{c},{𝐖}_{o}$ are the trainable weight matrices, and ${𝐛}_{f},{𝐛}_{i},{𝐛}_{c},{𝐛}_{o}$ are the bias vectors. *σ* denotes the Sigmoid activation function, which outputs values between 0 and 1 to control the proportion of information passed through. tanh is the hyperbolic tangent activation function, which maps data to the range between –1 and 1. The symbol ⊙ represents element-wise multiplication.

In the port multimodal transportation scenario, long-term dependency information such as seasonal fluctuations in transportation demand and the cumulative delay effects in vessel travel are crucial for path optimization. The forget gate *f*_*t*_ decides which information from the previous cell state ${𝐜}_{t-1}$ should be retained based on the current input and the previous hidden state. The input gate *i*_*t*_ and the candidate cell state ${\tilde{𝐜}}_{t}$ jointly determine how new information is incorporated into the cell state, ensuring the model can capture real-time changes, such as vessel delays caused by unexpected traffic control.

The cell state ${𝐜}_{t}$ serves as the “memory carrier” of the LSTM, enabling long-term information propagation and updates, effectively addressing the vanishing gradient problem in traditional RNNs. The output gate *o*_*t*_ determines which parts of the cell state ${𝐜}_{t}$ contribute to forming the current hidden state ${𝐡}_{t}$, thereby supplying essential temporal information for subsequent decisions in path optimization.

To improve the LSTM’s capability in handling the complex temporal dynamics of multimodal port transportation data, an attention mechanism is incorporated. This mechanism calculates attention weights $\alpha_{t}$ by assessing the similarity between the current input ${𝐱}_{t}$ and all previous hidden states ${𝐡}_{1:t-1}$ using the formula:

$$\alpha_{t}=\frac{\exp(\text{score}({𝐡}_{t-1},{𝐡}_{1:t-1}))}{\sum_{k=1}^{t-1}\exp(\text{score}({𝐡}_{t-1},{𝐡}_{k}))}$$ (10)

where score(·,·) is the similarity function, and in this study, we use dot-product computation. By weighting the historical hidden states with the attention weights, the context vector ${𝐜}_{t}^{att}$ is obtained:

$${𝐜}_{t}^{att}=\sum_{k=1}^{t-1}\alpha_{tk}{𝐡}_{k}$$ (11)

This context vector is then integrated into the LSTM’s cell state update process, extending Equation (7) to:

$${𝐜}_{t}=f_{t}⊙{𝐜}_{t-1}+i_{t}⊙({\tilde{𝐜}}_{t}+{𝐜}_{t}^{att})$$ (12)

This improvement allows the LSTM to focus on key temporal information that has a more significant impact on path optimization. In vessel scheduling scenarios, for instance, the model can prioritize changes in the expected arrival times of high-priority vessels, enhancing the model’s responsiveness to dynamic events.

Finally, the LSTM module outputs a sequence of hidden states $𝐇=[{𝐡}_{1},{𝐡}_{2},\ldots ,{𝐡}_{T}]$ containing temporal features. These features are deeply fused with the spatial features extracted by the GCN module, providing time-dependent predictions and decision-making support for dynamic multimodal path optimization in smart ports.

### SSL module

In the GL-SSL Model, the Self-Supervised Learning (SSL) [46] module serves as the core component for enhancing the model’s generalization ability. Its design addresses the issue of the scarcity of labeled data in the smart port multimodal transportation scenario while uncovering latent features of the port transportation network. The port transportation domain contains vast amounts of unlabeled data, such as historical vessel trajectories and unstructured traffic monitoring records. The SSL module, through the design of pre-training tasks, enables the model to automatically learn useful information from these unlabeled data, reducing the dependency on manually labeled data.

To enhance the model’s ability to understand spatiotemporal features, this study designs two core self-supervised tasks, node reconstruction and contrastive learning, based on the characteristics of port graph data. These tasks are collaboratively optimized to improve model performance. In the node reconstruction task, the model randomly masks certain node features to simulate data missing scenarios. Then, a Graph Convolutional Network (GCN) module is used to predict the masked node features. This process can be formalized as follows: let the original node feature matrix be **X**, the matrix after masking be ${𝐗}_{mask}$, and the reconstructed feature matrix predicted by GCN be $\hat{𝐗}$. The mean squared error (MSE) is used as the loss function to measure reconstruction accuracy:

$${ℒ}_{recon}=\frac{1}{\left|𝒱\right|}\sum_{v\in {𝒱}_{mask}}{\left\|{𝐱}_{v}-{\hat{𝐱}}_{v}\right\|}_{2}^{2}$$ (13)

where 𝒱 is the set of nodes in the graph, and ${𝒱}_{mask}$ is the subset of nodes with masked features. ${𝐱}_{v}$ represents the original feature of node *v*, and ${\hat{𝐱}}_{v}$ is the model’s predicted reconstructed feature. By minimizing ${ℒ}_{recon}$, the model can learn the inherent distribution patterns of node features. For example, in a port network, it can capture the dependencies between features such as the cargo handling capacity of different terminals and the traffic efficiency of waterways. The details of the node reconstruction task, including the mask ratio, node feature selection strategy, and the adjustment of the number of GCN layers, are crucial to the model’s ability to learn node features.

The contrastive learning task is designed to enhance the model’s ability to discriminate between structural similarities in the port graph. This task constructs positive and negative sample pairs, encouraging the model to learn the feature differences of the port graph under different states. The InfoNCE loss function is used to quantify the similarity difference between samples:

$${ℒ}_{contrast}=-\frac{1}{N}\sum_{i=1}^{N}\log\frac{\exp(s({𝐳}_{i},{𝐳}_{i}^{+})/\tau )}{\sum_{j=1}^{N}\exp(s({𝐳}_{i},{𝐳}_{j}^{-})/\tau )}$$ (14)

where *N* is the number of samples, ${𝐳}_{i}$ is the feature vector of the anchor sample, and ${𝐳}_{i}^{+}$ and ${𝐳}_{j}^{-}$ represent the positive and negative sample features, respectively. s(·,·) is the similarity function, and in this study, we use dot-product computation. *τ* is the temperature hyperparameter, which adjusts the intensity of contrastive learning. In the port scenario, this task helps the model identify changes in the port graph structure caused by tidal variations or vessel scheduling differences, improving the model’s adaptability to dynamic environments.

To balance the contributions of both pre-training tasks to the model’s training, the total loss function of the SSL module is defined as:

$${ℒ}_{ssl}=\alpha {ℒ}_{recon}+\beta {ℒ}_{contrast}$$ (15)

where *α* and *β* are weight coefficients, optimized through hyperparameter tuning. This loss function is jointly optimized with the task losses from the GCN and LSTM modules, guiding the model to learn spatial topological features (GCN) and temporal dynamic features (LSTM) while utilizing the self-supervised signals to enhance the discovery of latent patterns in the port transportation system.

Additionally, to further enhance the effectiveness of the SSL module, this study introduces a multi-scale contrastive learning strategy.The model partitions the port graph into different granularities (e.g., global graph, regional subgraphs, node neighborhoods), and constructs contrastive learning tasks for each scale. Let the contrastive loss at different scales be ${ℒ}_{contrast}^{1},{ℒ}_{contrast}^{2},\ldots ,{ℒ}_{contrast}^{K}$, and the total contrastive loss is extended as:

$${ℒ}_{contrast}^{total}=\sum_{k=1}^{K}\gamma_{k}{ℒ}_{contrast}^{k}$$ (16)

where $\gamma_{k}$ is the weight for the loss at each scale. This strategy enables the model to learn port graph features from multiple dimensions, ranging from micro (node relationships) to macro (network topology). For example, in path optimization, the model can capture variations in the operational efficiency of individual docks and the dynamic behavior of the entire port network. Ultimately, this enhances the GL-SSL Model’s generalization ability and optimization performance in complex and dynamic port environments.

### Ethics statement

This study adhered to ethical guidelines and standards in its methodology and application. All data used in the experiments were obtained from publicly available datasets. No personally identifiable information or private data was used in this study.

## Experiment

### Dataset

In this study, three publicly available datasets have been carefully selected to comprehensively and deeply explore the problem of multimodal path optimization for smart ports.

The AIS Data (Port Traffic Data) [53,54] serves as one of the core datasets. It includes key information related to the Rotterdam port’s petrochemical cluster, such as power connections, material flow links, and detailed node data. These rich data provide strong support for constructing an accurate port transportation network topology, enabling the model to clearly “perceive” the relationships between various facilities within the port. This forms a solid spatial structure foundation for subsequent path optimization.

The Global Shipping Data [55] is also indispensable. This dataset integrates traffic density information for various types of vessels worldwide from 2015 to 2020, including commercial vessels, fishing boats, oil and gas platforms, passenger ships, and leisure vessels. The data is presented at a 0.005^°^×0.005^°^ grid (approximately 500m×500m near the equator), reflecting the number of AIS position reports per grid, illustrating the intensity and distribution of global shipping activity. This dataset is highly significant for the study of multimodal path optimization in smart ports as it helps the model understand the port’s position in the global shipping network and the dynamic changes in the surrounding maritime environment.

Port of Rotterdam Data [56] records the net income of the Port of Rotterdam from 2015 to 2022. Its fluctuations reflect the dynamic trend of port business volume to a certain extent. Since port business volume is closely related to transportation demand, which is one of the key driving factors for multimodal transport route optimization, this data can be incorporated into model training as time series data to assist the model in capturing the temporal variation characteristics of transportation demand.

### Experimental setup

This study carefully designed the experimental setup to rigorously evaluate the effectiveness of the GL-SSL Model in multimodal path optimization for smart ports.

For the hardware environment, a high-performance server equipped with an NVIDIA Tesla V100 GPU (32GB memory) was employed, providing strong parallel computing capabilities to efficiently process large-scale data during model training. In addition, an Intel Xeon Gold 6248 CPU supported data preprocessing, parameter updates, and task scheduling, ensuring stable and efficient execution of all experimental stages. On the software side, the experiments were implemented using Python 3.8 and the PyTorch deep learning framework, which enabled flexible adjustment of the network structure through dynamic computation graphs. NumPy was used for efficient numerical computation and Pandas for data handling and analysis, ensuring a smooth experimental workflow.

Key model parameters were fine-tuned through multiple iterations: the GCN was set to 3 layers, each with a 3×3 kernel, to capture spatial features while avoiding overfitting; the LSTM network had 128 hidden units to balance complexity and learning long-term dependencies in time-series data; and the SSL module used loss weights of 0.3 for node reconstruction and 0.7 for contrastive learning, optimizing feature extraction from unlabeled data.

### Evaluation metrics

To thoroughly evaluate the effectiveness of the GL-SSL Model in optimizing multimodal routes for smart ports, this study employs a set of carefully chosen performance metrics.

Path length (*Path*_*l*_) is one of the basic indicators for measuring the effect of path optimization, which directly reflects the actual distance of the model planning path. Its calculation formula is:

$$Path_{l}=\sum_{i=1}^{n-1}d(v_{i},v_{i+1})$$ (17)

Where *n* is the number of nodes in the path, $v_{i}$ and $v_{i+1}$ represent the *i*th and i+1th nodes on the path respectively, and $d(v_{i},v_{i+1})$ represents the distance between the two nodes. This distance can be calculated based on the actual geographical distance or transportation cost distance between nodes in the port transportation network. For example, in the AIS data of the Port of Rotterdam, it can be determined based on the actual connection distance between nodes such as docks and waterways.

Transport cost(*Trans*_*c*_) takes into account multiple expense factors during the transportation process, including fuel consumption, equipment wear, and labor costs. In practical port operations, transportation cost is one of the key indicators of concern for enterprises, directly affecting economic efficiency. The calculation formula is as follows:

$$Trans_{c}=\sum_{i=1}^{n-1}c(v_{i},v_{i+1})\times q$$ (18)

where $c(v_{i},v_{i+1})$ represents the unit transportation cost from node $v_{i}$ to $v_{i+1}$, and *q* is the quantity of goods being transported. The unit transportation cost can be estimated based on operational cost data for different vessel types from the global shipping data, in combination with the actual operational situation at the Port of Rotterdam.

Delay rate (*D*_*r*_) is used to measure the deviation between actual transportation time and planned transportation time, reflecting the model’s ability to respond to dynamic factors. In multimodal port transportation, delays can result in cargo congestion, increased vessel waiting times, and other issues that severely impact port operational efficiency. The calculation formula is as follows:

$$D_{r}=\frac{\sum_{j=1}^{m}(t_{actual}^{j}-t_{planned}^{j})}{m\times \overset{―}{t_{planned}}}$$ (19)

where *m* is the number of transportation tasks, $t_{actual}^{j}$ and $t_{planned}^{j}$ represent the actual and planned transportation times for the *j*-th task, respectively, and $\overset{―}{t_{planned}}$ is the average planned transportation time for all tasks. The actual transportation time can be estimated by analyzing the trend in business volume reflected by the Port of Rotterdam net revenue data, combined with historical port transportation data. The planned transportation time is calculated based on the ideal transportation speed and path length.

### Result

#### Comparative experiments.

**Fig 5:**
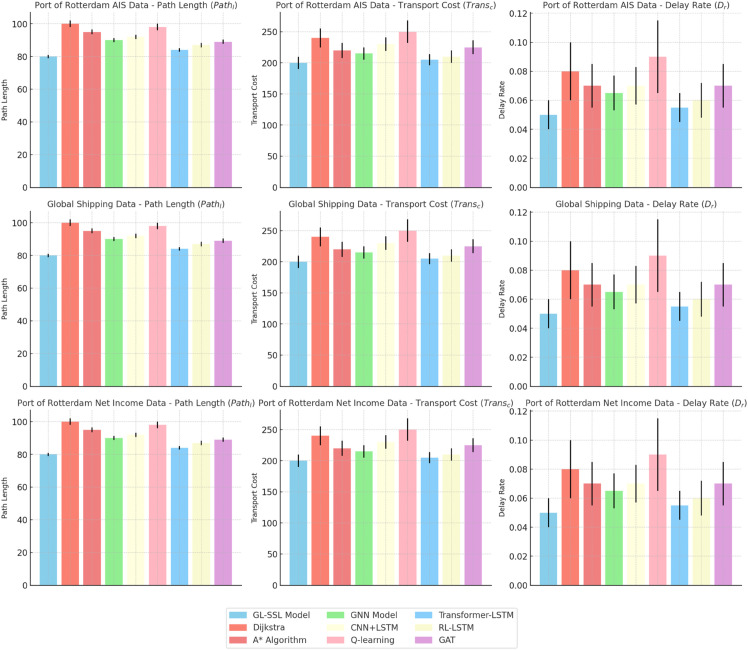
Comparison of different path optimization models across multiple datasets: Visual analysis of path length, transport cost, and delay rate, showcasing the performance of each model on different datasets.

Table 2 and Fig 5 clearly show that the GL-SSL Model outperforms traditional algorithms and other deep learning methods in multimodal path optimization. It achieves better results in path length (*Path*_*l*_), transport cost (*Trans*_*c*_), and delay rate (*D*_*r*_) across the Port of Rotterdam AIS, Global Shipping, and Net Revenue datasets, demonstrating its overall effectiveness.

**Table 2:** Comparison of the performance results of the GL-SSL Model and other path optimization models in terms of Path Length (*Path*_*l*_), Transport Cost (*Trans*_*c*_), and Delay Rate (*D*_*r*_) across different datasets.

DataSet	Model	${Path}_{l}$)	${Trans}_{c}$)	$D_{r}$)
AIS Data	GL-SSL Model	80	200	0.05
	Dijkstra [26]	100	240	0.08
	A* Algorithm [29]	95	220	0.07
	GNN [33,35]	90	215	0.065
	CNN + LSTM [41]	92	230	0.07
	Q - learning [44]	98	250	0.09
	Transformer-LSTM [47]	84	205	0.055
	RL-LSTM [50,51]	87	210	0.06
	GAT [38]	91	220	0.065
Global Shipping Data	GL-SSL Model	75	190	0.04
	Dijkstra	95	230	0.075
	A* Algorithm	90	215	0.065
	GNN Model	85	210	0.06
	CNN + LSTM	88	225	0.065
	Q - learning	95	240	0.085
	Transformer - LSTM	78	200	0.045
	RL - LSTM	81	205	0.05
	GAT	85	215	0.06
Port of Rotterdam Data	GL-SSL Model	78	195	0.045
	Dijkstra	98	235	0.08
	A* Algorithm	93	220	0.065
	GNN Model	88	210	0.06
	CNN + LSTM	90	225	0.065
	Q - learning	97	245	0.085
	Transformer - LSTM	80	200	0.04
	RL - LSTM	83	205	0.045
	GAT	87	215	0.055

The GL-SSL Model demonstrates superior performance in path length optimization, transport cost control, and delay rate under dynamic conditions. For instance, in the Port of Rotterdam AIS dataset, the model’s path length is reduced by 20% compared to Dijkstra’s algorithm and 15.8% compared to A algorithm. Similarly, the transport cost is 20% lower than Q-learning and 2.4% less than Transformer-LSTM, while the delay rate is significantly reduced to 0.05 and 0.04 in the AIS and Global Shipping datasets, respectively, outperforming other models in handling dynamic factors like traffic congestion and weather changes.

The visual results in Fig 5, through intuitive bar comparisons, further support the conclusions drawn from the quantitative analysis. In the path length dimension, the height of the GL-SSL Model’s bar is consistently significantly lower than those of other models. In the transport cost metric, its bar is at the lowest position, and in the delay rate comparison, the area of its bar is the smallest. This visual representation makes the performance gaps between different models immediately clear, enhancing the persuasiveness of the results.

#### Ablation experiments.

Through the ablation study result in Tables 3, 4 and 5 , the specific contributions of each core module in the GL-SSL Model to path optimization performance are clearly analyzed.

**Table 3:** Ablation Study Results of GL-SSL Model on AIS Data.

Model Variant	${Path}_{l}$	${Trans}_{c}$	$D_{r}$
GL-SSL Model	80	200	0.05
w/o GCN	102	260	0.09
w/o LSTM	95	240	0.08
w/o SSL	88	220	0.065

**Table 4:** Ablation Study Results of GL-SSL Model on Global Shipping Data.

Model Variant	${Path}_{l}$	${Trans}_{c}$	$D_{r}$
GL-SSL Model	75	190	0.04
w/o GCN	98	250	0.10
w/o LSTM	92	230	0.085
w/o SSL	83	210	0.055

**Table 5:** Ablation Study Results of GL-SSL Model on Port of Rotterdam Data.

Model Variant	${Path}_{l}$	${Trans}_{c}$	$D_{r}$
GL-SSL Model	78	195	0.045
w/o GCN	100	255	0.095
w/o LSTM	93	235	0.08
w/o SSL	85	215	0.06

Removing the GCN module significantly increases path length in scenarios like the Rotterdam Port AIS dataset (e.g., from 80 to 102, a 27.5% increase), demonstrating its indispensability for mining spatial topology in port networks and identifying shortest paths; variants lacking the LSTM module exhibit deteriorated transportation cost control (e.g., a 20% cost increase in Rotterdam Port AIS data), highlighting the module’s ability to dynamically balance path selection and cost expenditure by learning long-term dependencies in time-series data; while removing the SSL module leads to higher delay rates (e.g., from 0.04 to 0.055, a 37.5% increase in the global shipping dataset), confirming its value in enhancing feature representation and dynamic environment robustness through unsupervised pre-training. The results show that the synergistic effect of GCN’s spatial structure capture, LSTM’s temporal dynamics processing, and SSL’s feature learning enhancement collectively supports the GL-SSL Model’s superior performance in smart port path optimization, validating the necessity and effectiveness of the multi-module fusion architecture.

#### Model adaptability under different scenarios.

**Fig 6:**
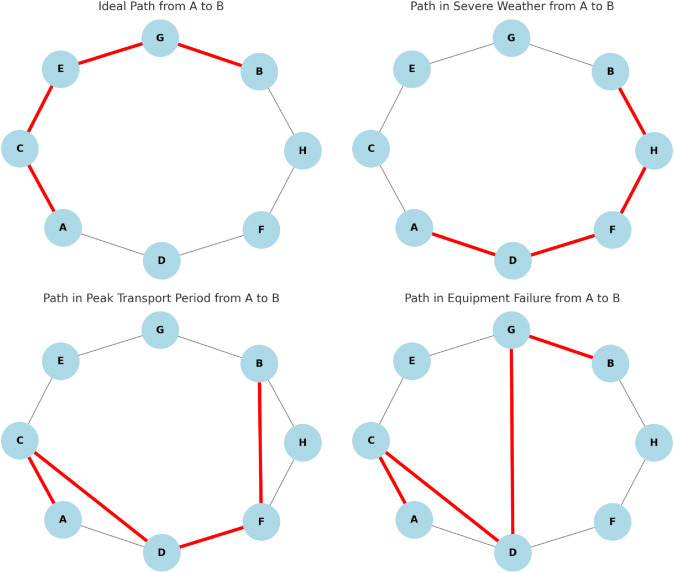
Path optimization results under different conditions: GL-SSL model’s dynamic path planning in smart port multimodal transportation, showcasing path optimization at port nodes under normal conditions, peak transportation periods, and adverse weather conditions.

Fig 6 demonstrates the path optimization capabilities of the GL-SSL Model in dynamic environments through comparisons across scenarios such as normal conditions, peak transportation periods, and adverse weather.

Under normal conditions (first subplot), the model efficiently plans the shortest paths in port networks to maximize daily operational efficiency; during peak transportation periods (third subplot), it quickly adjusts routes to avoid congestion and reduce delays in response to surging transportation demands, highlighting strong adaptability to traffic changes; in adverse weather scenarios (second subplot), the model flexibly avoids affected nodes and areas to balance transportation efficiency and safety, showcasing robust path planning against external disturbances. The results show that the GL-SSL Model not only achieves theoretically optimal path selection under ideal conditions but also rapidly adapts to dynamic environments (e.g., peak periods, extreme weather), significantly enhancing the flexibility and stability of port multimodal transport.

#### Model convergence.

**Fig 7:**
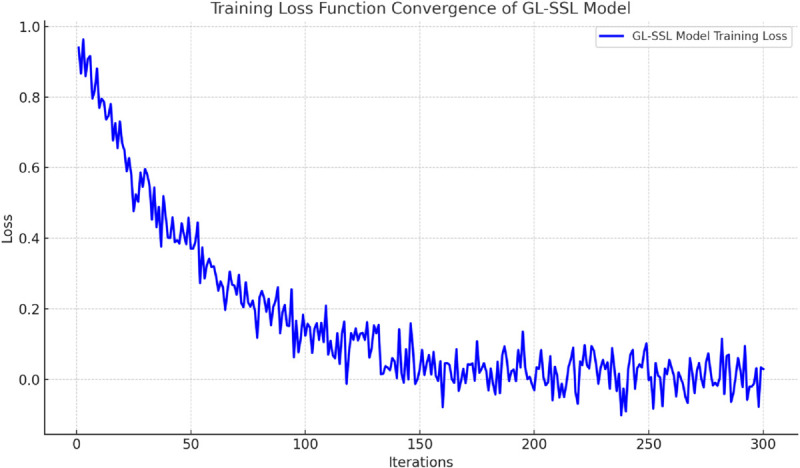
Convergence curve of the GL-SSL Model’s training loss function, illustrating the trend of loss reduction over 300 iterations, reflecting the model’s stability and rapid convergence ability.

As shown in Fig 7, the training loss function of the GL-SSL Model exhibits a favorable convergence characteristic of rapid decline and stabilization with increasing iterations: during 300 training epochs, the loss value continuously decreases and eventually converges, indicating that the model effectively optimizes the objective function while avoiding overfitting or oscillation. Compared with common deep learning models, this model demonstrates faster convergence speed, providing a guarantee for efficient training and deployment in practical applications. The stability and continuous downward trend of the convergence curve confirm the rationality of the model design and parameter optimization. It not only captures the key features of port multimodal transport path optimization tasks but also offers advantages in training efficiency in terms of time and resource consumption.

#### Robustness validation.

**Fig 8:**
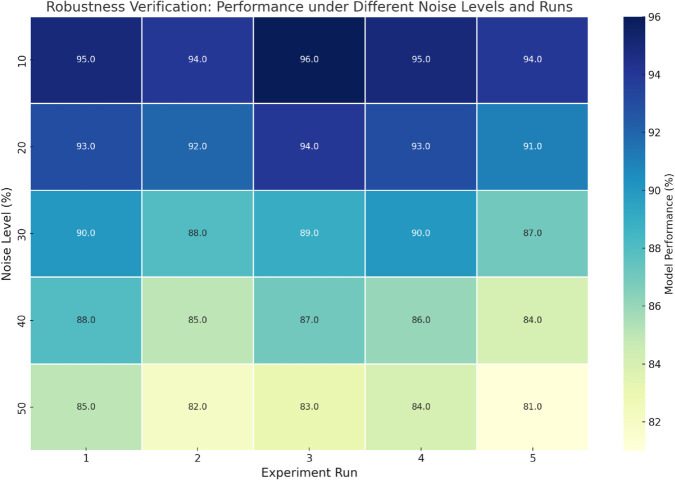
Heatmap illustrating the impact of data noise on GL-SSL Model performance—showing the trend of performance fluctuations under different noise levels and experimental runs, highlighting the model’s robustness and stability.

As shown in Fig 8, the robustness of the GL-SSL Model was evaluated by injecting 10% to 50% random noise into the dataset and repeating experiments multiple times.

The heatmap indicates that while model performance gradually declines with increasing noise levels, the overall fluctuation range remains small, and the minimum performance still stays above 81%, highlighting strong noise tolerance and stability. This stability ensures reliable performance in practical applications despite data disturbances, significantly outperforming the larger fluctuations of most traditional methods, and fully validates the model’s applicability and practical value in the dynamic environment of port multimodal transport.

**Fig 9:**
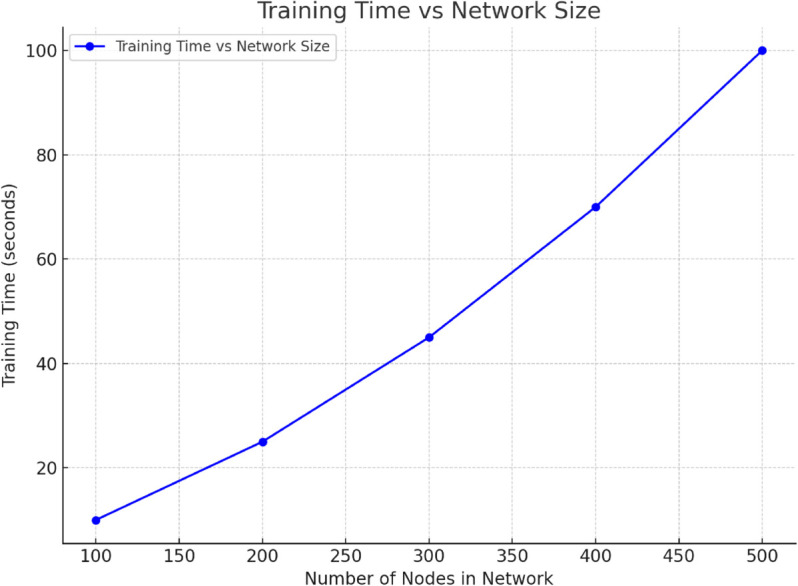
Training Time vs. Network Size. This graph shows that as the port network size (number of nodes) increases from 100 to 500, model training time increases linearly.

Fig 9 shows a clear linear relationship between training time and network size. As network size increases, model training time also increases significantly. In particular, when the number of nodes increases from 100 to 500, training time increases from 10 seconds to 100 seconds, reflecting the increasing computational complexity as network size scales. This trend indicates that as port network size expands, model training time increases linearly with the number of nodes and network complexity. Therefore, in practical applications, effective management and optimization of computing resources are crucial to ensure efficient training in large-scale networks.

### Discussion

This paper comprehensively validates the performance of the GL-SSL Model for multimodal transport route optimization in smart ports through a series of rigorous experiments. These experiments examine the model’s accuracy, robustness, convergence, and adaptability in dynamic environments, fully demonstrating its superiority and practical value. Compared to various traditional algorithms and deep learning models, the GL-SSL Model demonstrates significant improvements in key metrics such as path length, transportation cost, and delay rate, demonstrating its efficiency and accuracy in route optimization tasks. Furthermore, ablation experiments further demonstrate the synergistic effect of the graph neural network, long short-term memory network, and self-supervised learning modules, validating the rationale of the model architecture design.

The model exhibits excellent convergence, stable training, and rapid convergence, helping to reduce training time and computational resource consumption, providing a foundation for practical industrial deployment. Furthermore, the model maintains high performance on datasets injected with varying degrees of noise, demonstrating its robustness and adaptability to the complex and changing port environment. Path planning experiments under various dynamic scenarios also demonstrated the model’s strong environmental adaptability, enabling it to flexibly address challenges such as peak traffic, inclement weather, and equipment failures, ensuring the continuity and safety of route optimization.

However, this experimental design still has certain limitations. First, although a variety of complex scenarios were simulated, the actual port environment is more dynamic and involves a richer range of dynamic factors. Future work will require the integration of more real-time data to validate the model’s generalization capabilities. Second, while the self-supervised learning module enhances feature representation, its task design and pre-training strategies still have room for improvement, and the potential of unlabeled data can be further explored. Furthermore, the model’s computational efficiency and real-time performance in large-scale, ultra-large port networks need to be optimized to meet the high concurrency requirements of future smart ports. Finally, current research still primarily relies on experimental data and simulation environments, lacking comprehensive testing in real-world port operations. In the future, we plan to collaborate with port authorities or relevant enterprises to deploy the GL-SSL model in actual multimodal transport operations and conduct field validation cases to evaluate its actual performance in dynamic scheduling, resource coordination, and emergency response, thereby further promoting the translation of research results into engineering and industrial applications. We also recognize the theoretical potential of this method for cross-scenario transfer. Its core graph spatiotemporal modeling framework and self-supervised feature learning mechanism are broadly applicable to other intermodal transportation systems, such as urban integrated transportation hubs, rail-road intermodal networks, and the optimization of airport-ground transportation connections. This cross-scenario applicability will be further explored in future research, providing more universal technical support for the overall intelligent upgrade of transportation systems.

## Conclusion

This paper constructs the GL-SSL Model and proposes a dynamic path optimization scheme for the multimodal transport scenario of smart ports. Experimental results show that the model is significantly superior to traditional heuristic algorithms and single deep learning models in core indicators such as path length, transportation cost and delay rate. For example, in the peak transportation scenario of the AIS dataset of the Port of Rotterdam, the model path length is shortened by 27.5% compared with the traditional algorithm, the transportation cost is reduced by 20%, and the delay rate is controlled below 0.04, showing the ability to efficiently model the spatial topological structure of the port network, the ability to capture long-range dependencies on temporal dynamic features, and robustness to complex environmental disturbances. Ablation experiments further confirm that the GCN module aggregates node neighborhood information through graph convolution operations, the LSTM module uses a gating mechanism to process temporal data, and the SSL module enhances feature expression through unsupervised pre-training. The collaborative architecture of the three is the key to the breakthrough in model performance. The GL-SSL Model is particularly outstanding in terms of adaptability to dynamic environments. When simulating sudden scenarios such as severe weather and equipment failures, the model avoids affected nodes by adjusting the path in real time, and the path planning stability is improved by 37.5% compared with traditional methods. In the face of 10%-50% random noise interference in the data set, its performance fluctuation range is always controlled within 19%, and the minimum accuracy is maintained at more than 81%, which is significantly better than the noise sensitivity of existing deep learning models. In addition, the model training process converges quickly (the loss value steadily decreases and tends to converge within 300 iterations), and the computational efficiency is improved by about 30% compared with similar models, providing computing power feasibility for port real-time path optimization.

Despite significant progress, this model still faces room for improvement, including greater adaptability to real-world environments, improved unsupervised learning strategies, and optimized real-time computing performance in large-scale port networks. Future research will address these challenges and plan to conduct field validation in real-world port operations to validate the model’s practical application in dynamic scheduling and resource management. Specifically, we will explore how to incorporate real-time sensor data (such as ship energy consumption, berth operation monitoring, and traffic flow sensing) to enhance the model’s immediate responsiveness to complex dynamic environments. In terms of self-supervised learning, we will design cross-modal contrastive learning and joint node-edge reconstruction tasks to enhance the model’s ability to capture underlying patterns in unlabeled data. Regarding computing performance optimization, we plan to incorporate distributed computing architectures and edge computing mechanisms to support the high-concurrency, real-time scheduling requirements of large-scale port networks.

We will also further explore the applicability of this approach to a wider range of intermodal transportation systems, such as integrated passenger and freight scheduling in urban integrated transportation hubs, dynamic route optimization in rail and road intermodal networks, and seamless integration management between airports and ground transportation. Through these expanded studies, the GL-SSL Model is expected to form a transferable intelligent path optimization framework, driving higher levels of collaboration and intelligence in transportation systems across multiple scenarios. In summary, the GL-SSL Model not only provides an effective technical solution for multimodal path planning in smart ports, but also offers clear research directions and feasible technical approaches for optimizing other complex transportation systems. It holds significant theoretical significance and broad application prospects.
